# Supplementary material for: Pyruvate Dehydrogenase Kinase 1 inhibition mediated oxidative phosphorylation enhancement in cartilage promotes osteoarthritis progression
Source: BMC Musculoskelet Disord. 2023 Jul 20;24:597. doi: 10.1186/s12891-023-06585-6 (PMC10357736; doi:10.1186/s12891-023-06585-6)
Supplement: Supplementary file 1 — Additional file 1. [file 12891_2023_6585_MOESM1_ESM.zip › KEGG.Gsea.1653622432464/KEGG_RENAL_CELL_CARCINOMA.html]

Details for gene set KEGG\_RENAL\_CELL\_CARCINOMA[GSEA]

|  || Dataset | 55457\_collapsed\_to\_symbols.cls.cls #OA\_versus\_normal.cls.cls #OA\_versus\_normal\_repos |
| Phenotype | cls.cls#OA\_versus\_normal\_repos |
| Upregulated in class | normal |
| GeneSet | KEGG\_RENAL\_CELL\_CARCINOMA |
| Enrichment Score (ES) | -0.34709382 |
| Normalized Enrichment Score (NES) | -1.4198914 |
| Nominal p-value | 0.02970297 |
| FDR q-value | 0.20477262 |
| FWER p-Value | 0.989 |
Table: GSEA Results Summary

  

Fig 1: Enrichment plot: KEGG\_RENAL\_CELL\_CARCINOMA      
 Profile of the Running ES Score & Positions of GeneSet Members on the Rank Ordered List

  

| SYMBOL | TITLE | RANK IN GENE LIST | RANK METRIC SCORE | RUNNING ES | CORE ENRICHMENT || 1 | RBX1 | ring-box 1 [Source:HGNC Symbol;Acc:HGNC:9928] | 348 | 0.724 | 0.0046 | No |
| 2 | MAPK3 | mitogen-activated protein kinase 3 [Source:HGNC Symbol;Acc:HGNC:6877] | 757 | 0.579 | -0.0018 | No |
| 3 | ELOB | elongin B [Source:HGNC Symbol;Acc:HGNC:11619] | 933 | 0.531 | 0.0079 | No |
| 4 | AKT1 | AKT serine/threonine kinase 1 [Source:HGNC Symbol;Acc:HGNC:391] | 1192 | 0.478 | 0.0087 | No |
| 5 | CRK | "CRK proto-oncogene, adaptor protein [Source:HGNC Symbol;Acc:HGNC:2362]" | 2018 | 0.360 | -0.0398 | No |
| 6 | PIK3CB | "phosphatidylinositol-4,5-bisphosphate 3-kinase catalytic subunit beta [Source:HGNC Symbol;Acc:HGNC:8976]" | 2186 | 0.340 | -0.0379 | No |
| 7 | VEGFC | vascular endothelial growth factor C [Source:HGNC Symbol;Acc:HGNC:12682] | 2239 | 0.335 | -0.0273 | No |
| 8 | CRKL | "CRK like proto-oncogene, adaptor protein [Source:HGNC Symbol;Acc:HGNC:2363]" | 2283 | 0.329 | -0.0162 | No |
| 9 | TGFB1 | transforming growth factor beta 1 [Source:HGNC Symbol;Acc:HGNC:11766] | 2490 | 0.303 | -0.0190 | No |
| 10 | HGF | hepatocyte growth factor [Source:HGNC Symbol;Acc:HGNC:4893] | 2547 | 0.297 | -0.0103 | No |
| 11 | ARNT2 | aryl hydrocarbon receptor nuclear translocator 2 [Source:HGNC Symbol;Acc:HGNC:16876] | 3512 | 0.201 | -0.0766 | No |
| 12 | CUL2 | cullin 2 [Source:HGNC Symbol;Acc:HGNC:2552] | 3660 | 0.189 | -0.0798 | No |
| 13 | PAK1 | p21 (RAC1) activated kinase 1 [Source:HGNC Symbol;Acc:HGNC:8590] | 3861 | 0.169 | -0.0880 | No |
| 14 | VHL | von Hippel-Lindau tumor suppressor [Source:HGNC Symbol;Acc:HGNC:12687] | 4142 | 0.143 | -0.1035 | No |
| 15 | GRB2 | growth factor receptor bound protein 2 [Source:HGNC Symbol;Acc:HGNC:4566] | 5697 | 0.016 | -0.2239 | No |
| 16 | FH | fumarate hydratase [Source:HGNC Symbol;Acc:HGNC:3700] | 5729 | 0.013 | -0.2258 | No |
| 17 | RAF1 | "Raf-1 proto-oncogene, serine/threonine kinase [Source:HGNC Symbol;Acc:HGNC:9829]" | 6187 | -0.019 | -0.2605 | No |
| 18 | TGFB3 | transforming growth factor beta 3 [Source:HGNC Symbol;Acc:HGNC:11769] | 6293 | -0.027 | -0.2675 | No |
| 19 | EGLN1 | egl-9 family hypoxia inducible factor 1 [Source:HGNC Symbol;Acc:HGNC:1232] | 6364 | -0.032 | -0.2716 | No |
| 20 | ELOC | elongin C [Source:HGNC Symbol;Acc:HGNC:11617] | 6556 | -0.046 | -0.2845 | No |
| 21 | RAP1A | "RAP1A, member of RAS oncogene family [Source:HGNC Symbol;Acc:HGNC:9855]" | 6580 | -0.048 | -0.2841 | No |
| 22 | MAPK1 | mitogen-activated protein kinase 1 [Source:HGNC Symbol;Acc:HGNC:6871] | 6853 | -0.068 | -0.3024 | No |
| 23 | RAP1B | "RAP1B, member of RAS oncogene family [Source:HGNC Symbol;Acc:HGNC:9857]" | 6980 | -0.078 | -0.3088 | No |
| 24 | TGFB2 | transforming growth factor beta 2 [Source:HGNC Symbol;Acc:HGNC:11768] | 6985 | -0.079 | -0.3056 | No |
| 25 | SLC2A1 | solute carrier family 2 member 1 [Source:HGNC Symbol;Acc:HGNC:11005] | 7089 | -0.087 | -0.3098 | No |
| 26 | RAC1 | Rac family small GTPase 1 [Source:HGNC Symbol;Acc:HGNC:9801] | 7192 | -0.096 | -0.3136 | No |
| 27 | NRAS | "NRAS proto-oncogene, GTPase [Source:HGNC Symbol;Acc:HGNC:7989]" | 7300 | -0.102 | -0.3175 | No |
| 28 | MET | "MET proto-oncogene, receptor tyrosine kinase [Source:HGNC Symbol;Acc:HGNC:7029]" | 7322 | -0.104 | -0.3145 | No |
| 29 | PIK3CD | "phosphatidylinositol-4,5-bisphosphate 3-kinase catalytic subunit delta [Source:HGNC Symbol;Acc:HGNC:8977]" | 7405 | -0.111 | -0.3161 | No |
| 30 | PAK5 | p21 (RAC1) activated kinase 5 [Source:HGNC Symbol;Acc:HGNC:15916] | 7558 | -0.124 | -0.3225 | No |
| 31 | PIK3CA | "phosphatidylinositol-4,5-bisphosphate 3-kinase catalytic subunit alpha [Source:HGNC Symbol;Acc:HGNC:8975]" | 7733 | -0.136 | -0.3301 | No |
| 32 | PAK2 | p21 (RAC1) activated kinase 2 [Source:HGNC Symbol;Acc:HGNC:8591] | 7762 | -0.139 | -0.3262 | No |
| 33 | PIK3CG | "phosphatidylinositol-4,5-bisphosphate 3-kinase catalytic subunit gamma [Source:HGNC Symbol;Acc:HGNC:8978]" | 7953 | -0.152 | -0.3343 | No |
| 34 | HRAS | "HRas proto-oncogene, GTPase [Source:HGNC Symbol;Acc:HGNC:5173]" | 7977 | -0.153 | -0.3294 | No |
| 35 | AKT3 | AKT serine/threonine kinase 3 [Source:HGNC Symbol;Acc:HGNC:393] | 8008 | -0.156 | -0.3249 | No |
| 36 | SOS2 | SOS Ras/Rho guanine nucleotide exchange factor 2 [Source:HGNC Symbol;Acc:HGNC:11188] | 8058 | -0.160 | -0.3217 | No |
| 37 | ARAF | "A-Raf proto-oncogene, serine/threonine kinase [Source:HGNC Symbol;Acc:HGNC:646]" | 8092 | -0.163 | -0.3172 | No |
| 38 | BRAF | "B-Raf proto-oncogene, serine/threonine kinase [Source:HGNC Symbol;Acc:HGNC:1097]" | 8171 | -0.169 | -0.3159 | No |
| 39 | PAK3 | p21 (RAC1) activated kinase 3 [Source:HGNC Symbol;Acc:HGNC:8592] | 8266 | -0.176 | -0.3155 | No |
| 40 | VEGFB | vascular endothelial growth factor B [Source:HGNC Symbol;Acc:HGNC:12681] | 8673 | -0.206 | -0.3380 | Yes |
| 41 | TGFA | transforming growth factor alpha [Source:HGNC Symbol;Acc:HGNC:11765] | 8748 | -0.212 | -0.3345 | Yes |
| 42 | GAB1 | GRB2 associated binding protein 1 [Source:HGNC Symbol;Acc:HGNC:4066] | 8786 | -0.215 | -0.3279 | Yes |
| 43 | PIK3R3 | phosphoinositide-3-kinase regulatory subunit 3 [Source:HGNC Symbol;Acc:HGNC:8981] | 8801 | -0.217 | -0.3195 | Yes |
| 44 | PIK3R5 | phosphoinositide-3-kinase regulatory subunit 5 [Source:HGNC Symbol;Acc:HGNC:30035] | 8804 | -0.217 | -0.3102 | Yes |
| 45 | FLCN | folliculin [Source:HGNC Symbol;Acc:HGNC:27310] | 8848 | -0.221 | -0.3038 | Yes |
| 46 | EGLN3 | egl-9 family hypoxia inducible factor 3 [Source:HGNC Symbol;Acc:HGNC:14661] | 8965 | -0.229 | -0.3028 | Yes |
| 47 | PAK6 | p21 (RAC1) activated kinase 6 [Source:HGNC Symbol;Acc:HGNC:16061] | 9068 | -0.237 | -0.3004 | Yes |
| 48 | ARNT | aryl hydrocarbon receptor nuclear translocator [Source:HGNC Symbol;Acc:HGNC:700] | 9427 | -0.267 | -0.3166 | Yes |
| 49 | ETS1 | "ETS proto-oncogene 1, transcription factor [Source:HGNC Symbol;Acc:HGNC:3488]" | 9795 | -0.301 | -0.3320 | Yes |
| 50 | MAP2K2 | mitogen-activated protein kinase kinase 2 [Source:HGNC Symbol;Acc:HGNC:6842] | 9932 | -0.315 | -0.3288 | Yes |
| 51 | PAK4 | p21 (RAC1) activated kinase 4 [Source:HGNC Symbol;Acc:HGNC:16059] | 10004 | -0.322 | -0.3202 | Yes |
| 52 | MAP2K1 | mitogen-activated protein kinase kinase 1 [Source:HGNC Symbol;Acc:HGNC:6840] | 10197 | -0.340 | -0.3202 | Yes |
| 53 | EGLN2 | egl-9 family hypoxia inducible factor 2 [Source:HGNC Symbol;Acc:HGNC:14660] | 10212 | -0.342 | -0.3063 | Yes |
| 54 | KRAS | "KRAS proto-oncogene, GTPase [Source:HGNC Symbol;Acc:HGNC:6407]" | 10544 | -0.377 | -0.3156 | Yes |
| 55 | CREBBP | CREB binding protein [Source:HGNC Symbol;Acc:HGNC:2348] | 10881 | -0.414 | -0.3236 | Yes |
| 56 | PIK3R2 | phosphoinositide-3-kinase regulatory subunit 2 [Source:HGNC Symbol;Acc:HGNC:8980] | 10956 | -0.425 | -0.3107 | Yes |
| 57 | VEGFD | vascular endothelial growth factor D [Source:HGNC Symbol;Acc:HGNC:3708] | 11095 | -0.443 | -0.3021 | Yes |
| 58 | PIK3R1 | phosphoinositide-3-kinase regulatory subunit 1 [Source:HGNC Symbol;Acc:HGNC:8979] | 11099 | -0.443 | -0.2829 | Yes |
| 59 | PTPN11 | protein tyrosine phosphatase non-receptor type 11 [Source:HGNC Symbol;Acc:HGNC:9644] | 11260 | -0.465 | -0.2749 | Yes |
| 60 | EP300 | E1A binding protein p300 [Source:HGNC Symbol;Acc:HGNC:3373] | 11741 | -0.540 | -0.2887 | Yes |
| 61 | EPAS1 | endothelial PAS domain protein 1 [Source:HGNC Symbol;Acc:HGNC:3374] | 11758 | -0.542 | -0.2662 | Yes |
| 62 | HIF1A | hypoxia inducible factor 1 subunit alpha [Source:HGNC Symbol;Acc:HGNC:4910] | 11833 | -0.556 | -0.2476 | Yes |
| 63 | SOS1 | SOS Ras/Rac guanine nucleotide exchange factor 1 [Source:HGNC Symbol;Acc:HGNC:11187] | 12041 | -0.603 | -0.2372 | Yes |
| 64 | PDGFB | platelet derived growth factor subunit B [Source:HGNC Symbol;Acc:HGNC:8800] | 12389 | -0.704 | -0.2334 | Yes |
| 65 | VEGFA | vascular endothelial growth factor A [Source:HGNC Symbol;Acc:HGNC:12680] | 12629 | -0.813 | -0.2164 | Yes |
| 66 | CDC42 | cell division cycle 42 [Source:HGNC Symbol;Acc:HGNC:1736] | 12681 | -0.852 | -0.1830 | Yes |
| 67 | JUN | "Jun proto-oncogene, AP-1 transcription factor subunit [Source:HGNC Symbol;Acc:HGNC:6204]" | 12813 | -0.994 | -0.1496 | Yes |
| 68 | PGF | placental growth factor [Source:HGNC Symbol;Acc:HGNC:8893] | 12850 | -1.059 | -0.1060 | Yes |
| 69 | RAPGEF1 | Rap guanine nucleotide exchange factor 1 [Source:HGNC Symbol;Acc:HGNC:4568] | 12860 | -1.098 | -0.0585 | Yes |
| 70 | AKT2 | AKT serine/threonine kinase 2 [Source:HGNC Symbol;Acc:HGNC:392] | 12896 | -1.404 | 0.0003 | Yes |
Table: GSEA details [plain text format]

  

Fig 2: KEGG\_RENAL\_CELL\_CARCINOMA      
 Blue-Pink O' Gram in the Space of the Analyzed GeneSet

  

Fig 3: KEGG\_RENAL\_CELL\_CARCINOMA: Random ES distribution      
 Gene set null distribution of ES for **KEGG\_RENAL\_CELL\_CARCINOMA**
